# Model-Based Dose Identification of Dalbavancin for Long-Term Suppressive Outpatient Treatment of Ventricular Assist Device Infections

**DOI:** 10.3390/antibiotics13111103

**Published:** 2024-11-20

**Authors:** Ute Chiriac, Uwe Liebchen, Otto Roman Frey, Heike Lanzinger, Sabrina Klein, Torsten Hoppe-Tichy, Matthias Karck, Anna Meyer, Benedict Morath

**Affiliations:** 1 Hospital Pharmacy, Heidelberg University Hospital, Im Neuenheimer Feld 670, 69120 Heidelberg, Germany; 2 Department of Anaesthesiology, University Hospital, Ludwig Maximilians University of Munich, Marchioninistraße 15, 81377 Munich, Germany; 3 Hospital Pharmacy, General Hospital Heidenheim, Schlosshausstrasse 100, 89522 Heidenheim, Germany; 4 Department of Infectious Diseases, Medical Microbiology and Hygiene, Medical Faculty Heidelberg, Heidelberg University, Im Neuenheimer Feld 324, 69120 Heidelberg, Germany; 5 University Hospital Heidelberg, Im Neuenheimer Feld 324, 69120 Heidelberg, Germany; 6 Department of Cardiac Surgery, Heidelberg University Hospital, Im Neuenheimer Feld 410, 69120 Heidelberg, Germany

**Keywords:** dalbavancin, ventricular assist device, population pharmacokinetics, therapeutic drug monitoring, long-term treatment

## Abstract

Increasing evidence suggests that dalbavancin is an effective long-term treatment for ventricular assist device (VAD) infections, with various prolonged dosing regimens currently in use. This retrospective study aimed to assess dalbavancin pharmacokinetics in VAD patients and identify optimal, feasible dosing regimens for long-term suppressive outpatient therapy. Data from Heidelberg University Hospital’s VAD register were analyzed using non-linear mixed-effects modeling for pharmacokinetic analysis and dosing simulations (Lixoft^®^). The probability of target attainment (PTA) and cumulative fraction of response (CFR) were calculated for different protein-binding scenarios considering the minimum inhibitory concentration (MIC) distribution of *Staphylococcus aureus*. Using data from 13 patients with 38 blood samples, a two-compartment model best described the dalbavancin pharmacokinetics, with a typical value for clearance of 0.050 L/h, central volume of distribution of 6.5 L, and peripheral volume of 15.4 L. No covariates significantly improved the model fit. The observed protein binding varied between 96 and 98%. Dosing simulations demonstrated that 1500 mg every 3 weeks ensured the target attainment for stasis at MIC values of 0.125 mg/L (PTA ≥ 90%) up to a protein binding of 99%. Considering the CRF, longer dosing intervals up to 5 weeks might be possible. Depending on individual MICs and protein binding, a dalbavancin regimen of 1500 mg every 3 to 5 weeks therefore appears to be a valuable option for outpatient therapy of VAD infections. Therapeutic drug monitoring should be considered to manage inter-individual variability and to support clinicians in long-term treatments of subacute and chronic infections.

## 1. Introduction

Dalbavancin is a lipoglycopeptide antibiotic exhibiting efficacy against *Enterococcus* spp., *Staphylococcus* spp. (including MRSA), and *Streptococcus* spp. that is used for the treatment of acute bacterial skin and soft tissue infections (ABSSI) [1,2]. While currently approved for a two-week treatment of ABSSSI, its pharmacokinetic/pharmacodynamic (PK/PD) profile suggests potential for broader applications.

Dalbavancin has a high degree of protein binding of 93 to 99% and a unique half-life of 374 h [2,3]. The reported pharmacokinetic/pharmacodynamic (PK/PD) target is the free area under the concentration curve (*f*AUC)/minimum inhibitory concentration (MIC) with *f*AUC/MIC > 27.1 for stasis, >53.3 for log-1 kill, and >111.1 for log-2 kill for infection with *Staphylococcus aureus* [4].

Because of its long half-life, the labeled dose of 1500 mg administered once intravenously is sufficient to cover a treatment period of at least fourteen days, making it a promising treatment option for infections requiring prolonged treatment [2]. Therefore, alternative dosing regimens have been investigated in addition to the labeled treatment of ABSSI [5,6]. A recent expert statement reported the administration of 1500 mg twice within 4 weeks to be sufficient to cover a treatment period of 6 weeks [6]. Interestingly, some small cohorts of special patient populations, such as patients with infected ventricular assist devices (VADs), have been treated with dalbavancin as a long-term suppression therapy with different dosing regimens of 500 mg weekly and 1500 mg biweekly [7,8]. VAD patients represent a unique population and are prone to persistent and relapsing infections, as the source cannot be easily controlled [7,9]. Consequently, effective long-term dosing regimens need to guarantee adequate exposure while balancing the risk of accumulation. As only the unbound concentration is pharmacologically active, it seems reasonable to consider dalbavancin’s in vivo plasma protein binding when developing such long-term dosing regimens. To facilitate implementation, such regimens should be suitable for outpatient practice and reduce healthcare utilization.

The increasing use of dalbavancin in off-label settings for specialized patient populations such as endocarditis, osteomyelitis, spondylodiscitis, or prosthetic vascular graft infections highlights the need for further investigation to optimize its use in clinical practice [9,10]. By investigating alternative dosing strategies, dalbavancin utilization could also be optimized in off-label scenarios.

Thus, this study aimed to assess dalbavancin pharmacokinetics in patients with persistent VAD infection and identify suitable dosing regimens for long-term suppressive outpatient therapy, considering the potential impact of different protein-binding scenarios on target attainment.

## 2. Results

### 2.1. Patient Population

In total, 13 patients with long-term dalbavancin suppression therapy were identified in the VAD register. Patient demographic and clinical characteristics are summarized in Table 1. All patients were male and had an implanted Heart Mate III (Abbott). All patients underwent surgical source control, in combination with vacuum-assisted closure therapy and intravenous antimicrobial therapy, before the start of suppression therapy with dalbavancin.

The patients received a repetitive dalbavancin dose of 1500 mg at day 1 and day 8. Only one patient received a reduced dose of 1000 mg because of a CrCl < 30 mL/min. Dosing cycles were repeated every 6 weeks throughout the period of suppressive therapy. The mean ± SD dalbavancin total concentration at the end of infusion was 218.4 ± 65.9 mg/L after the first dose (*f*c_day1_ = 6.3 ± 2.7). By the end of the first week, the mean ± SD trough level was 41.1 ± 11.1 mg/L (*f*c_day8_ = 1.3 ± 0.6), and it further decreased to 13.2 ± 9.9 mg/L after 6 weeks. The observed protein binding varied between 96 and 98%. In all cases, the driveline was identified as the source of infection, with *S. aureus* detected in 12 cases and methicillin-resistant *S. aureus* (MRSA) in one case. Five patients had a concomitant bloodstream infection. The MIC was determined of a representative infection isolate in 10 cases before the start of dalbavancin therapy (3 × 0.047 mg/L, 5 × 0.064 mg/L, 1 × 0.094 mg/L, and 1 × 0.125 mg/L).

### 2.2. Model Building

**Figure 1:**
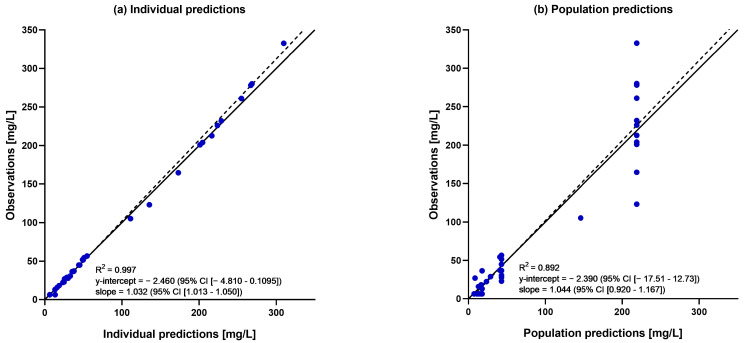
Goodness-of-fit plots illustrating (**a**) individual predictions and (**b**) population predictions vs. observed dalbavancin concentrations.

**Figure 2:**
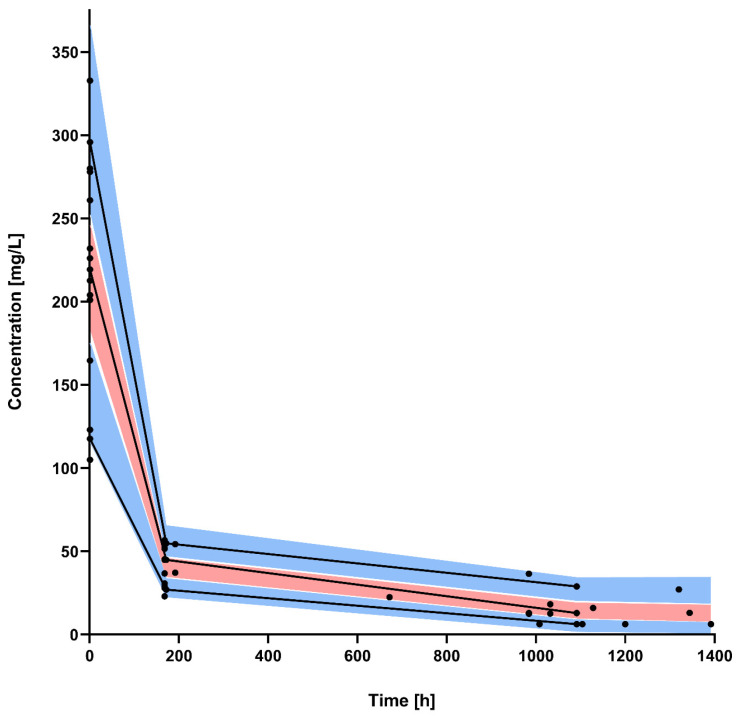
Visual predictive check for the final population pharmacokinetic model. Black dots are the observed dalbavancin concentrations; black lines represent the median, 10th, and 90th percentiles of the observed values; and shaded areas are the prediction intervals for the median (red central area) and 10th and 90th percentiles (light blue lower and upper areas).

A population pharmacokinetic model was developed for the total dalbavancin serum concentrations. A two-compartment model with fixed intercompartmental clearance (Q), based on a previous reported value, best described the data showing the lowest Akaike information criterion (AIC) (AIC of one-, two-, and three-compartment models: 383.08, 330.36, 343.09; Table S1) and the best predictive performance [11]. The linear regression of the observed versus model-predicted concentrations showed a good fit for both the population (R^2^ = 0.892) and the individual estimates (R^2^ = 0.997) (Figure 1). Trends were observed for certain covariates, such as body weight, BSA, and age, but no covariate relationships for any of the model parameters reached statistical significance. Detailed results and testing procedures are provided in the Supplementary Material (Tables S2–S4, Figure S1). The parameter estimates of the final model are summarized in Table 2. All estimates were accurate, with relative standard errors (RSEs) less than 30%, except for the residual error (b). The results of bootstrap medians and 95% confidence intervals were consistent and confirmed the reliability of the parameter and the random effect estimates. The MAX/MIN eigenvalue was 3.84, and shrinkage values are available in Figure S2. The visual predictive check (VPC) plot showed the good predictive performance of the model (Figure 2). Individual PK results obtained by Monolix for the PK model are shown in Table S5.

### 2.3. Dosing Simulations

**Figure 3:**
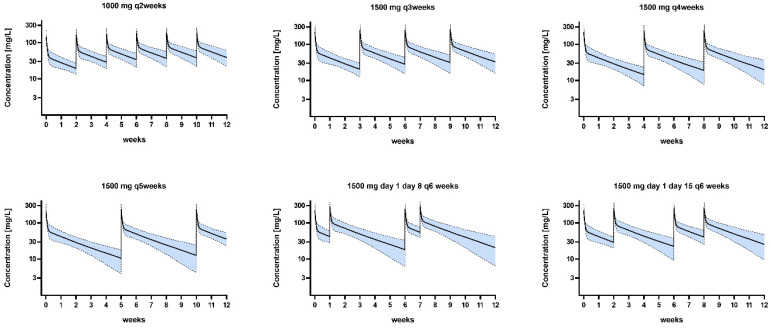
Concentration–time curves of explored dosing regimens in this study population. Presented as median concentration. The dashed lines are the 5th and 95th percentiles of simulated concentrations.

**Figure 4:**
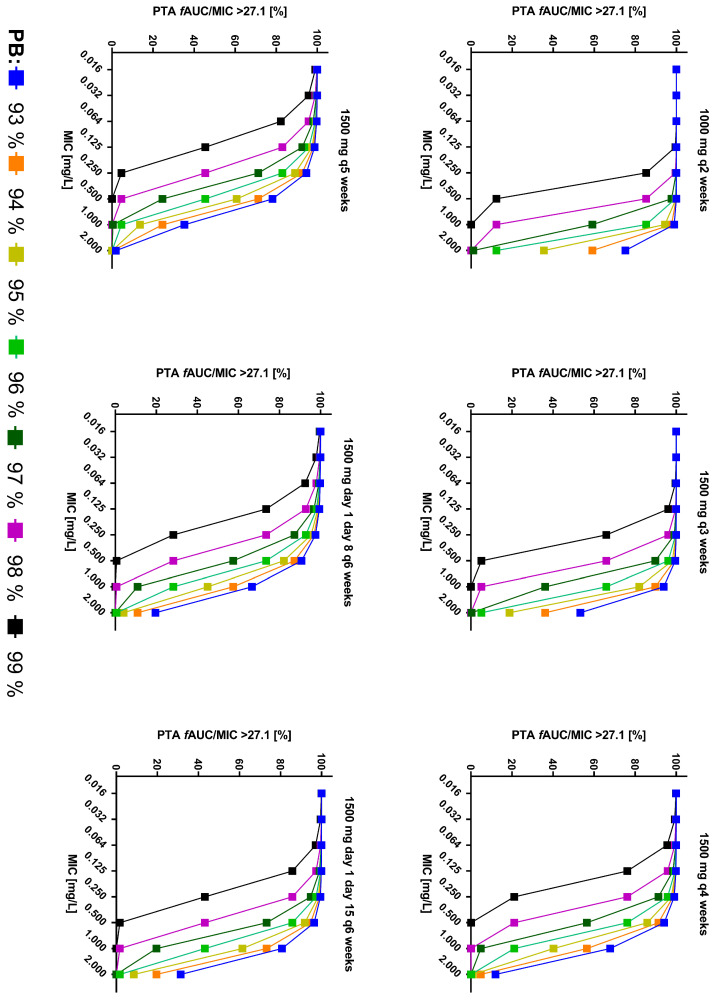
Probability of target attainment (PTA) in % to reach *f*AUC24 h/MIC > 27.1 for stasis vs. MIC values for different dosing regimens and protein bindings. ***f*AUC:** Free area under the concentration curve; **MIC:** Minimum inhibitory concentration; **PB:** Protein binding; **PTA:** Probability of target attainment.

Dosing simulations using the final model developed in this study are shown in Figure 3. These simulations revealed a target attainment ≥ 90% for stasis across all dosing regimens, considering the labeled protein binding of 93% and an epidemiological cut-off (ECOFF) for *S. aureus* according to EUCAST of 0.125 mg/L. The probabilities of target attainment (PTAs) for stasis are illustrated in Figure 4. Higher protein binding levels as observed in this study expectedly led to substantially lower PTA values. In a scenario of 99% protein binding, the ECOFF was only achieved with a dosing regimen of 1000 mg q2 weeks and 1500 mg q3 weeks (Figure 4).

**Figure 5:**
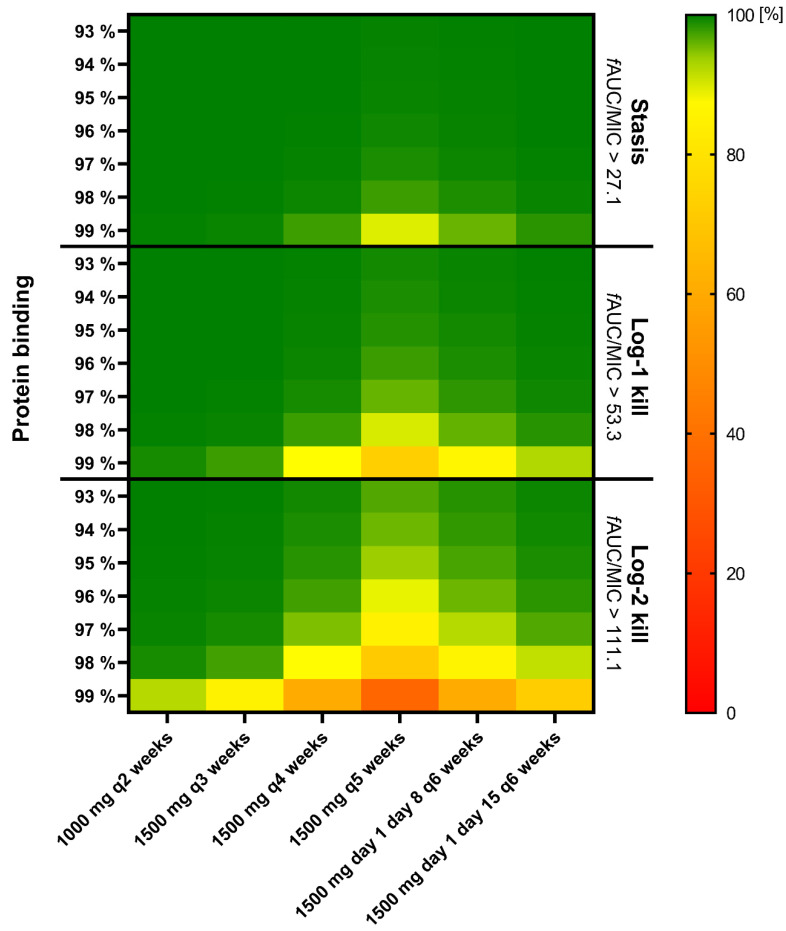
Cumulative fraction of response of various dalbavancin regimens against MIC distribution of *S. aureus* according to EUCAST at three PK/PD targets [12]. ***f*AUC:** Free area under the concentration curve|**MIC:** Minimum inhibitory concentration.

Considering the cumulative fraction of response (CFR), a dose of 1500 mg dalbavancin ensured a desirable target attainment up to 4 weeks at a PK/PD target of *f*AUC24h/MIC > 27.1 (stasis) and up to 3 weeks at that of >53.3 (log−1 kill) in a scenario of 99% protein binding (Figure 5 and Figure S3). With 98% protein binding, as observed in this study population, 1500 mg dalbavancin extended the desirable target attainment up to 5 weeks at a PK/PD target of *f*AUC24h/MIC > 27.1, up to 4 weeks at that of >53.3, and up to 3 weeks at that of >111.1 (Figure 5 and Figure S3).

## 3. Discussion

In this population, a PK analysis of dalbavancin concentrations in patients with persistent VAD infection with different reported dosage regimes for long-term suppressive therapy was conducted. The PTA analysis indicated that 1500 mg dalbavancin every 3 weeks achieved PTA for stasis at a MIC of 0.125 mg/L up to a protein binding of 99%. Considering the CFR, even longer intervals up to 5 weeks might be possible depending on individual protein binding, which was generally higher in this patient population than reported in the label.

The population PK of dalbavancin was investigated in two large phase II/III clinical trials involving ABSSI patients and real-life patients with bone and prosthetic infections [11,13,14]. Buckwalter et al. and Cojutti et al. reported clearance (CL) values of 0.057 L/h and 0.041 L/h, respectively, using a two-compartment model [11,13], while Carrothers et al. used a three-compartment model and reported a CL of 0.053 L/h [14]. The CL estimate of 0.050 L/h in this study aligns closely with these findings. In contrast, in this study a higher population VD for dalbavancin was observed (21.9 L vs. 15.0–16.7 L [11,13,14]), which may be attributed to the physiologic properties of VAD patients (e.g., fluid status). A high interindividual variability in pharmacokinetic parameters and protein binding was observed within this patient cohort. Potential clinical reasons for this variation might lie in pathophysiological changes in these special patients caused by underlying heart failure (e.g., impaired and unstable renal function, altered hemodynamics, fluid overload) potentially affecting dalbavancin exposure [15,16,17]. These findings align with previous studies reporting significant interindividual variability in dalbavancin serum concentrations associated with body surface area (BSA), weight, creatinine clearance (CrCl), and serum albumin as potential covariates [11,13,14]. Nevertheless, although tested, PK variability was not explained by any of these covariates in this study, probably due to the limited size of our population

From a PK/PD point of view, dalbavancin concentrations in our study population exceeded an *f*AUC/MIC of 27.1 for stasis with an applied dalbavancin dosing regimen of 1500 mg on day 1 and day 8, repeated every 6 weeks, in all but one patient at the end of the intended dosing interval (Table S5). However, no MIC value was determined in this patient, and the current EUCAST MIC distribution suggests a rather low probability of a high MIC (i.e., 0.125 mg/L). Simulations of various dosing regimens indicate stasis might be achieved across all of the tested regimens for the labeled protein binding of 93% and an EUCAST breakpoint for *S. aureus* of 0.125 mg/L [12]. In contrast, a significant protein binding of 99% might result in non-target attainment with the currently recommended dosing regimens [3]. Therefore, the accurate measurement of unbound drug concentrations should be a key consideration in any future dose–response studies, especially clinical trials to address this question. Considering the protein binding in our study population (96–98%), the simulations demonstrated an extended optimal target attainment for stasis for up to 5 weeks after a 1500 mg dose of dalbavancin. This finding supports the idea that other regimens may achieve the desired pharmacokinetic target for stasis, providing flexibility in dosing schedules, better aligning with scheduled routine outpatient visits or patient preferences. Thus, dosing regimens of 1500 mg every 3 to 5 weeks could serve as a valuable and cost-effective approach for outpatient settings treating VAD patients with long-term dalbavancin therapy, depending on individual protein binding and the observed MIC value. The TDM of dalbavancin, including the measurement of the unbound fraction, appears to be a valuable approach in addressing any underlying pharmacokinetic interindividual variability, for which also suitable HPLC methods are described [3,6,18]. Although this strategy appears to be feasible, data are needed on the duration of suppression therapy and whether and when therapy can be stopped in patients whose source cannot easily be controlled.

There are several limitations to this study. This was a retrospective study, and the study size was relatively small due to the special patient population, which expectedly led to higher RSE values of the estimate of the error model. Furthermore, this may have hampered the identification of covariates explaining some of the observed variability. Consequently, the generalizability of our results is not necessarily supported. However, eigen-values and shrinkage are low (see electronic Supplement Figure S2), which reflects a low risk of overparameterization. In addition, the estimates are in line with previously published models and confirmed by the bootstrap analysis.

## 4. Materials and Methods

### 4.1. Study Design

The department of cardiac surgery at Heidelberg University Hospital cares currently for around 80 VAD patients. Those with relapsing VAD infection under oral long-term suppression therapy or intolerance to therapy (adverse drug events, non-adherence, drug–drug interaction) can be treated with dalbavancin outpatient therapy. All patients on dalbavancin treatment received regular laboratory tests and the bioanalysis of total and unbound dalbavancin serum concentrations [18]. Sampling was routinely performed on three occasions for each patient at day 1 (30 min after the end of the infusion), day 7 (before redosing), and day 42 (before redosing). Blood samples were centrifuged (5 min, 4000 rpm) immediately after collection and aliquoted into 2 mL propylene tubes (Eppendorf, Hamburg, Germany). Aliquots were stored at −20 °C within 60 min after sample collection for a maximum of 4 weeks until assay. Dalbavancin serum concentrations were measured using high-performance liquid chromatography–ultraviolet spectrometry (HPLC) as previously described from our study group [18]. To obtain the unbound fraction, samples were prepared by solid phase extraction using a Nanosep^®^ filter (Pall, New York, NY, USA) [18]. The limit of quantification (QL) was 12.5 mg/L for the total concentration and 1 mg/L for the unbound fraction [18].

The MIC was tested with the MIC strips test (Liofilchem, Roseto degli Abruzzi, Italy) and broth microdilution assay according to EUCAST standards and the manufacturer’s instructions [12]. All patients with VAD-associated infections were included in a register study after providing informed consent. A positive vote of the ethics committee of Heidelberg University Hospital was obtained before the start of the study (S-674/2022). For the purpose of this study, the register was queried for patients on dalbavancin long-term suppression therapy. Patients were eligible for analysis if they had a documented VAD-associated infection requiring long-term suppressive therapy and had received at least two repetitive administrations of dalbavancin aiming for long-term suppression. Clinical data and laboratory and microbiological results were extracted.

### 4.2. Population Pharmacokinetic Analysis

Non-linear mixed-effects modeling with the stochastic approximation expectation (SAEM) algorithm in the Monolix software (version2023R1; Lixoft, Antony, France) was used. Maximum likelihood estimation was utilized to compute the individual pharmacokinetic parameters. Conditional means and standard deviations of individual pharmacokinetic parameters were calculated with Markov chain Monte Carlo convergence assessment. For model fitting, the objective function value (OFV) defined as minus two logarithms of the likelihood, the Akaike information criterion (AIC), and Bayesian information criterion (BIC) were calculated with Monte Carlo importance sampling (N = 10,000). Different error models (i.e., constant, combined, proportional) were explored. Interindividual variability was assumed to be log-normally distributed. Covariates were evaluated by the Pearson correlation test and Wald test [19]. The physiologically plausible covariates BSA, albumin, body weight, age, creatinine, and CrCl were tested to explain interindividual pharmacokinetic variability by a stepwise forward approach following a backward elimination procedure. The inclusion of covariates was guided by a statistically significant improvement in the model fit (forward OFV > 3.84, *p* < 0.05; backward OFV > 6.63, *p* < 0.01) and reduction in variability components (lower variability indicates a better model).

The predictive performance of the models was assessed by appropriateness of the relative standard errors (RSE < 30%) and visual inspection of the goodness-of-fit plots, including observed versus predicted dalbavancin concentrations, shrinkage, and VPC with n = 10,000 simulations. A non-parametric bootstrap analysis for each parameter was conducted by 1000 bootstraps using resampling by means with the Rsmlx (R speaks Monolix) package of R to determine a 95% confidence interval (CI). All statistical analyses and plotting were performed using GraphPad Prism and R Core Team (R Foundation for Statistical Computing, Vienna, Austria). PK parameters in the population were estimated by using all data, replacing data below the quantification limit with the value QL/2 (14%) [20]. Data were tested for normality with the Shapiro–Wilk test and are presented with mean ± SD and median and interquartile range where appropriate. 

### 4.3. Dosing Simulations

The PK/PD simulations and PTA were performed using Simulx (version2023R1; Lixoft, Antony, France) with the final estimation parameters to compare different dosing regimens in this study population (1500 mg day 1 and 8 q6 weeks, 1500 mg day 1 and day 15 q6 weeks, 1000 mg q2 weeks, 1500 mg q3 weeks, 1500 mg q4 weeks, and 1500 mg q5 weeks). The concentrations for dalbavancin were simulated for 10,000 individuals. The PK/PD target attainment of *f*AUC_24 h_/MIC (>27.1, >53.3, and >111.1) was used to assess dalbavancin concentration and MIC values from 0.016 to 2 mg/L for *S. aureus* [12]. The definition of the dalbavancin PK/PD index dalbavancin varies from one study to another [9,21,22]. To use a conservative approach in suppressive therapy reflecting efficacy in the crucial phase before redosing, the AUC was calculated at the end of the dosing interval (last 24 h before redosing) [21]. The PTA was analyzed for different reported protein-binding scenarios [3], including the labeled protein binding of 93% [2] and the observed values of the VAD cohort. In addition, the cumulative fraction of response (CFR) for each dalbavancin regimen was assessed by evaluating the PTA against the dalbavancin MIC distribution according to EUCAST from 0.016 to 2 mg/L [12]. The PTA and CFR were defined as desirable when ≥90%.

## 5. Conclusions

This pilot study suggests that a dose of 1500 mg dalbavancin ensures the PK/PD target for stasis against *S. aureus* in patients with persistent VAD infections for 3 weeks and might be extended to even longer intervals up to 5 weeks, depending on individual MIC values and protein binding. This duration aligns well with practical outpatient visit schedules occurring approximately every 4 weeks. Prospective studies are warranted to confirm the effectiveness and safety of this approach in such patients. TDM should be considered to manage interindividual variability and may enable personalized dosing in this vulnerable patient cohort, which may require long-term suppressive therapy for several months.

## Figures and Tables

**Table 1:** Demographic data of patient population.

Patient Demographics	
Sex	
male (n)	13
female(n)	0
Age (years)	57 ± 9
Diabetes mellitus type II (n)	5/13
Dialysis	0
Non-ischemic cardiomyopathy (n)	12/13
Ischemic cardiomyopathy (n)	1/13
Coronary artery diseases (n)	4/13
**Laboratory**	**Measured at first administration**
Creatinine clearance (mL/min)	90.8 ± 34.73
C-reactive protein (mg/L)	7.4 ± 5.7
Leucocytes (/nL)	8.3 ± 1.0
Body surface area (m^2^)	1.95 ± 0.17
Body mass index (kg/m^2^)	26.9 ± 3.9
Weight (kg) (median (IQR))	76 kg (20 kg)
Albumin (mg/dL)	42.5 ± 5.7

Data are presented as mean (±standard deviation (SD)) or median (interquartile range (IQR)) for continuous variables and as numbers for dichotomous variables.

**Table 2:** Parameter estimates of the final model describing the pharmacokinetics of dalbavancin in serum and the results of the bootstrap analysis.

Parameter	Estimate	RSE [%]	Bootstrap [mean (CI 95%)]
**CL (L/h)**	0.050	6.83	0.050 (0.044–0.055)
**V1 (L)**	6.5	8.05	6.42 (5.65–7.31)
**Q (L/h)**	0.476	-	–
**V2 (L)**	15.4	12.3	14.99 (12.46–18.67)
**ω_CL_**	0.230	22.5	0.22 (0.054–0.292)
**ω_V1_**	0.260	28.4	0.217 (0.094–0.325)
**ω_V2_**	0.410	24.3	0.357 (0.151–0.520)
**b**	0.100	54.6	0.128 (0.058–0.202)

Abbreviations: **% RSE**: Relative standard error of the estimate|**b:** Residual error (proportional)|**CL:** Total body clearance for dalbavancin|**Q:** Inter-compartmental clearance|**V1:** Central volume of distribution|**V2**: Peripheral volume of distribution|**ω:** Interindividual variability|**95% CI**: The 95% confidence interval determined by log-likelihood profiling.

## Data Availability

Data are available upon reasonable request.

## Supplementary Materials

The following supporting information can be downloaded at: https://www.mdpi.com/article/10.3390/antibiotics13111103/s1, Figure S1: Correlation of covariates and estimates of the final model; Figure S2: Conditional distribution of the parameter estimates including low shrinkage values; Figure S3: Cumulative fraction of response of various dalbavancin regimens (against MIC distribution of *Staphylococcus aureus* according to EUCAST) at three PK/PD targets. [2]| ***f*AUC:** Free area under the concentration curve|**MIC:** Minimum inhibitory concentration; Table S1: Structural model building; Table S2: Pearson’s correlation test of the random effect versus covariates; Table S3: Covariate model building; Table S4: Correlation test of individual parameter versus covariates. Table S5: Pharmacokinetic properties of dalbavancin of the respective patients.
