# Supplementary material for: Model-Based Dose Identification of Dalbavancin for Long-Term Suppressive Outpatient Treatment of Ventricular Assist Device Infections
Source: Antibiotics (Basel). 2024 Nov 20;13(11):1103. doi: 10.3390/antibiotics13111103 (PMC11591545; doi:10.3390/antibiotics13111103)
Supplement: Supplementary file 1 [file antibiotics-13-01103-s001.zip › antibiotics-3290188-supplementary.pdf]

Model based dose identification of dalbavancin for long-term suppressive outpatient treatment of ventricular assist device-infections

Table S1: Structural model building.

	1-compartment	2-compartment	3-compartment
Parameterization	CL, V	CL, V1, V2, Qfixed	CL, V1, V2, V3, Q2, Q3
OFV (-2×log-likelihood)	373.08	316.94	317.09
AIC	383.08	330.36	343.09
BIC	385.9	334.31	350.44
BICc	389.12	338.60	357.95

Abbreviations: **BIC:** bayesian information criteria | **BICc**: corrected bayesian information criteria | **CL**: total body clearance for dalbavancin | **OFV**, objective function value | **Q**: inter-compartmental clearance | **V1:** central volume of distribution | **V2:** peripheral volume of distribution| **V3:** peripheral volume of distribution | **AIC:** Akaike information criteria.

Model based dose identification of dalbavancin for long-term suppressive outpatient treatment of ventricular assist device-infections

Covariate model building

Table S2: Pearson's correlation test of the random effect versus covariates.

Covariates	Eta_Cl		Eta_V1		Eta_V2	
	Coeff	p-value	Coeff	p-value	Coeff	p-value
Age	-0.29	3.29e-1	-0.13	6.77e-1	0.50	8.46e-1
Albumin	0.030	9.22e-1	-0.008	9.79e-1	-0.37	2.14e-1
BSA	0.065	8.32e-1	0.550	4.97e-2	0.34	2.5e-1
Creatinine-clearance	0.069	8.23e-1	-0.072	8.16e-1	-0.35	2.42e-1
Creatinine	0.14	6.40e-1	0.39	1.86e-1	0.15	6.31e-1
Weight	0.090	7.71e-1	0.58	3.65e-2	0.33	2.67e-1

The covariate with the highest correlation is considered for inclusion into the base structural model (forward inclusion).

For covariate modelling, continuous variables were log-transformed centered by the weighted mean using following equation using Body surface area (BSA) as an example:

$$\log(\theta_i) = \theta_{pop} + \beta_\theta \log\left(\frac{BSA_i}{BSA_{mean}}\right) + \eta_{\theta,i}$$

Where θ_i is the pharmacokinetic parameter for the *i*th individual and θ_{pop} is the population parameter estimate of the parameter θ. $\log\left(\frac{BSA_i}{BSA_{mean}}\right)$ is the log-transformed BSA of the *i*th individual normalized by the weighted mean of the population. $\eta_{\theta,i}$ is the deviation from the population value of θ for the *i*th individual and β_θ is a scaling exponent of the population value of θ.

Model based dose identification of dalbavancin for long-term suppressive outpatient treatment of ventricular assist device-infections

Table S3: Covariate model building.

	Base model	Covariate model	Covariate model	Covariate model
	CL, V1, V2, Qfixed	CL, V1, V2, Qfixed	CL, V1, V2, Qfixed	CL, V1, V2, Qfixed
Added covariate	-	logWeight on V1	logBSA on V1	logAge on V2
OFV (-2×log-likelihood)	316.94	311.25	311.76	312.39
AIC	330.36	327.25	327.76	328.39
BIC	334.31	331.77	332.28	332.9
BICc	338.60	336.06	336.57	337.2

Abbreviations: **BIC:** bayesian information criteria | **BICc**: corrected bayesian information criteria | **BSA:** body surface area | **CL**: total body clearance for dalbavancin | **OFV**, objective function value | **Q**: inter-compartmental clearance | **V1:** central volume of distribution | **V2:** peripheral volume of distribution| **V3:** peripheral volume of distribution | **AIC:** Akaike information criteria.

Table S4: Correlation test of individual parameter versus covariates.

	Statistics	p-value
Beta_V1_logtWeight	4.17	1.57e-3
Beta_V1_logtBSA	2.5	2.29e-2
Beta V2 logtAge	3.51	4.85e-3

Model based dose identification of dalbavancin for long-term suppressive outpatient treatment of ventricular assist device-infections

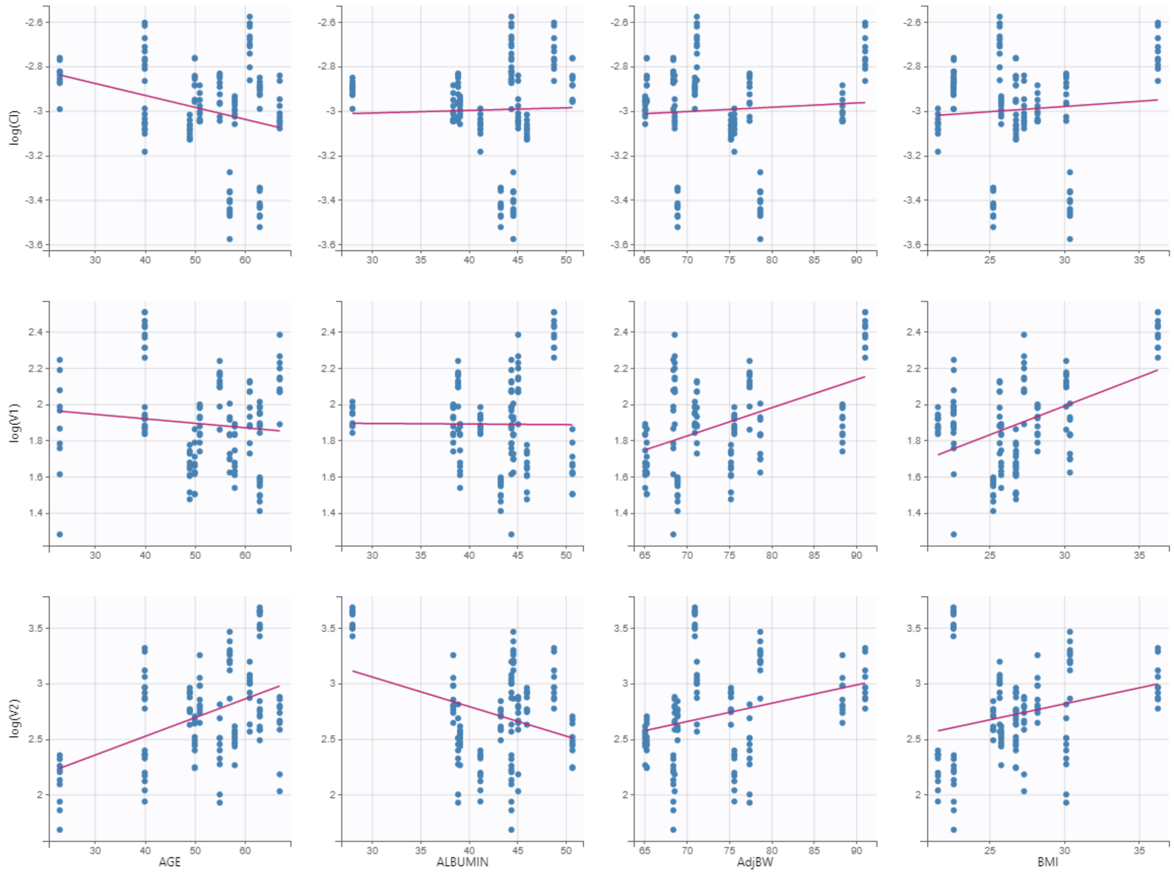

Figure S1: Correlation of covariates and estimates of the final model

Model based dose identification of dalbavancin for long-term suppressive outpatient treatment of ventricular assist device-infections

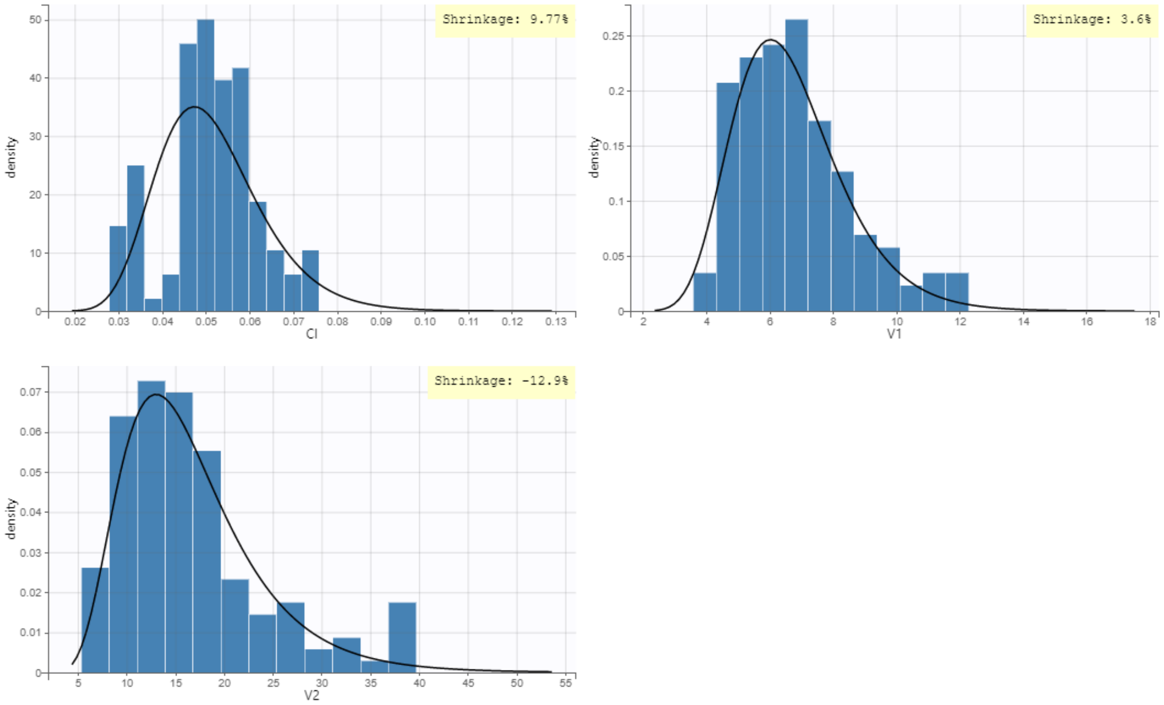

Figure S2: Conditional distribution of the parameter estimates including low shrinkage values.

Model based dose identification of dalbavancin for long-term suppressive outpatient treatment of ventricular assist device-infections

Table S5: Pharmacokinetic properties of dalbavancin of the respective patients

Patient number	CL (L/h)	V1 (L)	V2 (L)	MIC (mg/L)	Protein binding (%)	$AUC_{24\,h}$	$fAUC_{24\,h}$ /MIC
**1**	0.067	10.84	21.11	0.064	97.6	377	141
**2**	0.056	7.07	34.97	0.064	96.7	616	318
**3**	0.059	5.17	12.34	NA	97.0	216	26
**4**	0.033	6.23	24.73	NA	97.2	1138	127
**5**	0.046	5.25	16.48	0.094	98.1	547	110
**6**	0.052	8.68	13.65	NA	97.1	429	49
**7**	0.07	6.93	19.04	0.047	97.1	267	165
**8**	0.056	8.3	11.7	0.047	96.2	313	253
**9**	0.032	4.44	15.01	0.064	96.2	980	582
**10**	0.047	6.58	9.45	0.125	97.8	341	60
**11**	0.05	6.34	18.24	0.064	96.2	524	311
**12**	0.05	5.52	11.53	0.047	98.5	326	104
**13**	0.057	5.77	9.04	0.064	96.8	168	84

$AUC_{24\,h}$: Area under the concentration curve last 24 h before redosing **CL:** Total body clearance for dalbavancin | **$fAUC_{24\,h}$:** Free area under the concentration curve last 24 h before redosing | **MIC:** Minimum inhibitory concentration | **NA:** MIC not available, target calculated with *S. aureus* breakpoint 0.25 mg/L | **Q:** Inter-compartmental clearance | **V1:** Central volume of distribution | **V2**: peripheral volume of distribution |

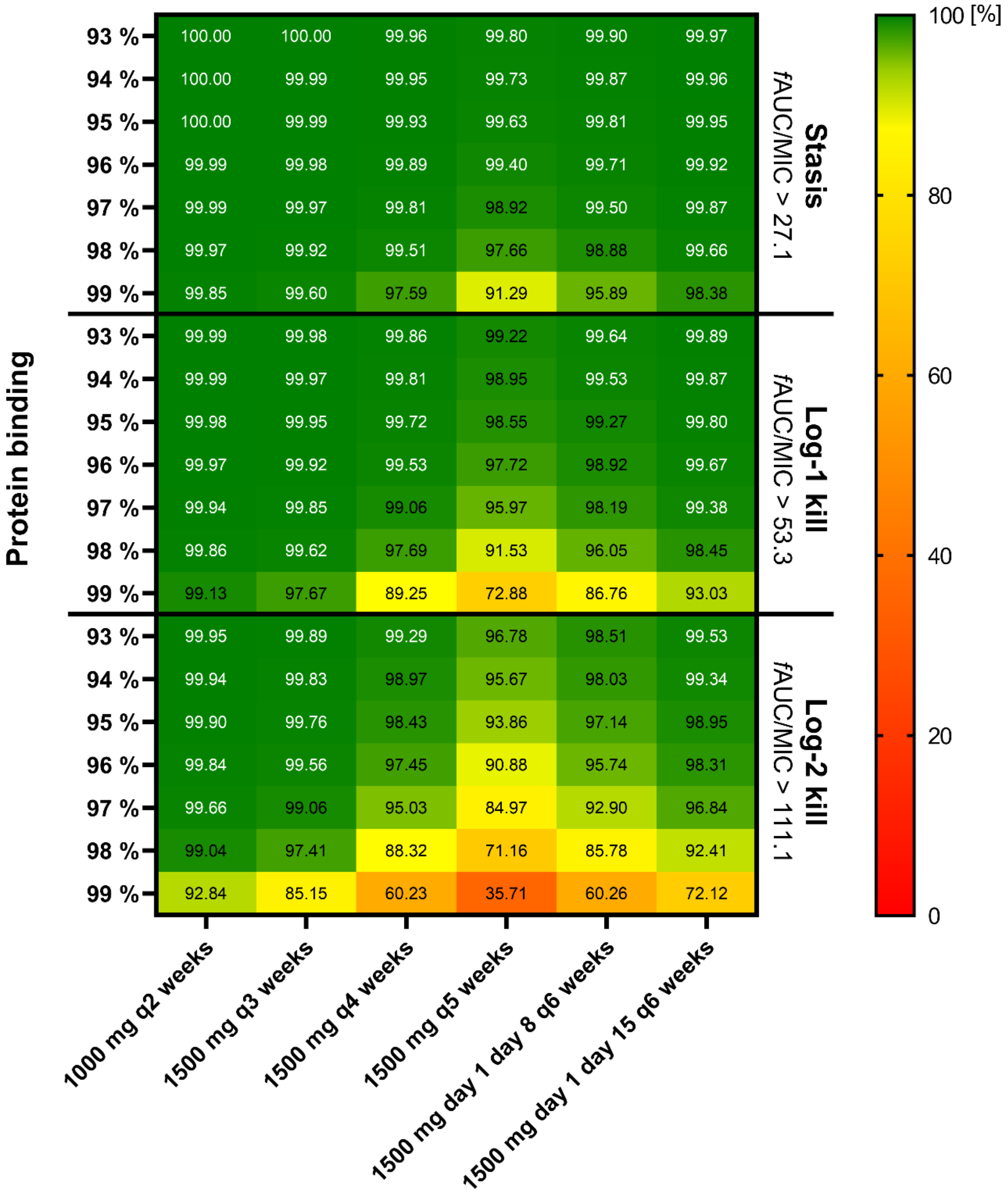

Figure S3: Cumulative fraction of response of various dalbavancin regimens (against MIC distribution of *Staphylococcus aureus* according to EUCAST) at three PK/PD targets. [2]| ***f*AUC:** Free area under the concentration curve | **MIC:** Minimum inhibitory concentration
